# Feeding of Whitefly on Tobacco Decreases Aphid Performance via Increased Salicylate Signaling

**DOI:** 10.1371/journal.pone.0138584

**Published:** 2015-09-18

**Authors:** Haipeng Zhao, Xiaoying Zhang, Ming Xue, Xiao Zhang

**Affiliations:** 1 Shandong Provincial Key Laboratory for Biology of Vegetable Diseases and Insect Pests, College of Plant Protection, Shandong Agricultural University, Tai’an, Shandong, China; 2 Kunming Institute of Botany, Chinese Academy of Sciences, Kunming, Yunnan, China; Zhejiang University, CHINA

## Abstract

**Background:**

The feeding of *Bemisia tabaci* nymphs trigger the SA pathway in some plant species. A previous study showed that *B*. *tabaci* nymphs induced defense against aphids (*Myzus persicae*) in tobacco. However, the mechanism underlying this defense response is not well understood.

**Methodology/Principal Findings:**

Here, the effect of activating the SA signaling pathway in tobacco plants through *B*. *tabaci* nymph infestation on subsequent *M*. *persicae* colonization is investigated. Performance assays showed that *B*. *tabaci* nymphs pre-infestation significantly reduced *M*. *persicae* survival and fecundity systemically in wild-type (WT) but not salicylate-deficient (NahG) plants compared with respective control. However, pre-infestation had no obvious local effects on subsequent *M*. *persicae* in either WT or NahG tobacco. SA quantification results indicated that the highest accumulation of SA was induced by *B*. *tabaci* nymphs in WT plants after 15 days of infestation. These levels were 8.45- and 6.14-fold higher in the local and systemic leaves, respectively, than in controls. Meanwhile, no significant changes of SA levels were detected in NahG plants. Further, biochemical analysis of defense enzymes polyphenol oxidase (PPO), peroxidase (POD), β-1,3-glucanase, and chitinase demonstrated that *B*. *tabaci* nymph infestation increased these enzymes’ activity locally and systemically in WT plants, and there was more chitinase and β-1, 3-glucanase activity systemically than locally, which was opposite to the changing trends of PPO. However, *B*. *tabaci* nymph infestation caused no obvious increase in enzyme activity in any NahG plants except POD.

**Conclusions/Significance:**

In conclusion, these results underscore the important role that induction of the SA signaling pathway by *B*. *tabaci* nymphs plays in defeating aphids. It also indicates that the activity of β-1, 3-glucanase and chitinase may be positively correlated with resistance to aphids.

## Introduction

The term induced plant resistance refers to biochemical, physiological, and developmental changes that take place in plants following stimuli, that can antagonize the settling, growth, development, and host-plant selection behavior of insects [1, 2]. These defenses limit damage caused by plant attackers and stimulate resistance to counter future challenges [3]. Plants have the ability to quickly and accurately perceive their biotic attackers and activate signaling pathways to ensure an effective spatial and temporal defense response [4]. The molecular mechanisms involved in plant defense are mediated mainly by the salicylic acid (SA) and jasmonic acid (JA)/ethylene (ET)-dependent signaling pathways [5]. There is accumulating evidence for defense-signaling pathways that are modulated after herbivore attack. In general, chewing herbivores have been largely associated with the jasmonate response [6, 7], while herbivory by phloem-sucking hemipterans, such as aphids and whiteflies, are often associated with the salicylate response [8–10].

SA is one of many phenolic compounds produced by plants. It is mostly known for its central role in defense responses, though it has been shown to regulate cell growth, stomatal aperture, respiration, seed germination, seedling development, thermotolerance, fruit yield, nodulation in legumes, and the expression of senescence-related genes [11, 12]. Studies on the SA signaling pathway have mainly focused on plant disease resistance reactions. SA regulates the expression of a wide array of defense responses including the pathogenesis-related (PR) proteins and defense enzymes, which have been shown to be important in both basal and resistance gene (R)-mediated biotrophic pathogen defense [13–15]. While studies on SA-mediated response in resistance to insect herbivores are relatively scarce, it was recently reported that SA is involved in the resistance of certain plants to phloem-feeding aphids [2, 16, 17].

The whitefly *Bemisia tabaci* Middle East-Asia Minor 1 (MEAM1) is an exotic pest insect that has a wide host range [18, 19]. The distinct competitive advantage of *B*. *tabaci* over the native population contributes to its outbreak [20]. The extreme polyphagy of *B*. *tabaci* creates an opportunity to engage in complicated interactions with numerous herbivorous arthropods in a variety of plant species and habitats [21]. Recently, studies of *B*. *tabaci* and its herbivorous competitors *Trichoplusia ni*, *Pieris rapae*, *Liriomyza trifolii*, and *Trialeurodes vaporariorum* have shown that the plants previously exposed to whitefly feeding to be less suitable for other herbivores, which subsequently showed behavioral differences (oviposition, feeding preferences) and reduced development, survival rates, and overall population growth [21–25].

The competition among herbivores may rely mostly on induced reactions in plants [26]. By identifying an attacker, plants are able to induce specific responses to herbivory. For instance, plants can discriminate between *B*. *tabaci* biotypes, *Macrosiphum euphorbiae* biotypes, and *Tetranychus urticae* lines [27–29]. These differences may result from differences in feeding activity and the saliva components of phloem feeders [30]. Recently, more studies have evaluated the defense of plants to phloem-feeding arthropod *B*. *tabaci*. For example, *B*. *tabaci* feeding induces expression of PR genes and other transcripts associated with salicylic acid (SA)-mediated signaling, similar to the host responses observed with pathogens or SA treatment [3, 31, 32]. In addition, increases in defense enzyme activities (chitinase, peroxidase or β-1,3-glucanase) have been observed after whitefly infestations [33–35]. However, fewer studies have focused on the role of induced resistance in competition between *B*. *tabaci* and other native insects, especially with respect to the SA-mediated defense response.

In our preliminary experiment, *B*. *tabaci* induced resistance to *M*. *persicae* in tobacco plants [36]. For this reason, it was here hypothesized that the defense response against *M*. *persicae* induced by *B*. *tabaci* will have a complicated relationship with the salicylate pathway. In this article, the potential association between the SA level and this induced resistance was studied to test this hypothesis. Utilizing transgenic NahG (suppresses SA accumulation) tobacco plants as a contrast, we aimed to answer the following three questions: (1) Is the defense of tobacco against subsequent *M*. *persicae* infestation different in WT and NahG plants pre-infested with *B*. *tabaci*? (2) Does the SA level have a direct relationship with the induced resistance to *M*. *persicae* in tobacco plants? (3) What defense enzymes contribute to this induced resistance against *M*. *persicae* in tobacco plants?

## Materials and Methods

### Tobacco plants

Tobacco (*Nicotiana tabacum* L. variety *Xanthi*-nc and NahG) seeds were supplied by Nanjing Agricultural University, Institute of Plant Protection, Department of Plant Pathology, Nanjing, Jiangsu Province, China. The seeds were sown in plastic trays (50 cm × 25 cm) containing garden soil. The plants were grown in growth chambers at 23 ± 2°C and a relative humidity of 75 ± 5%. Plants with two leaves were individually transplanted into plastic pots (10 cm deep, 12 cm in diameter) and placed in insect-proof screened cages (50 cm × 50 cm × 50 cm, 50 mesh). The plants were watered as needed and fertilized every 2 weeks at a rate of 0.05 g (N:P:K = 20:20:20) per plant and used for experiments when the plants had five leaves.

### Insect colonies

*Bemisia tabaci* Middle East-Asia Minor 1 (MEAM1) strain [37] was originally collected from cabbage and *M*. *persicae* was collected from tobacco plants grown at the Shandong Agricultural University Research Farm, Shandong Province, China. The insects were reared on tobacco plants for more than 30 generations. *B*. *tabaci* was identified based on the mitochondrial DNA COI gene sequence.

All of the pre-infestation and bioassay experiments were conducted in an artificial climate chamber (RTOP-D model, Top Instrument Corporation, Zhejiang, China) at 23 ± 2°C and 75 ± 5% RH, with a photoperiod of 12:12 (L:D) h.

### Whitefly infestation experiments

Pretreatment was performed in accordance with a method previously reported by Xue et al. [36]. Tobacco plants with five expanded leaves were placed in a nylon screen cage (50 cm × 50 cm × 50 cm) with ≈ 500 (± 10) whitefly adults (≈ 1:1 female:male) per plant. After 4 h of feeding, adult whiteflies were removed by aspiration to synchronize egg hatching and nymphal development.

### Effects of pre-infested tobacco plants on the survival and fecundity of aphids

Plants infested by whitefly nymphs were used after the nymphs reached the third instar (≈ 20 d). At this time plants had developed to seven to eight fully expanded leaves. There were ≈ 700–800 whitefly nymphs on each of the four bottom leaves (first to fourth), and the top five younger leaves (fifth to ninth) were whitefly free. The fourth leaf, which had 9–10 nymphs/cm^2^, and the seventh leaf, which had no nymphs, were here defined as local and systemic leaves, respectively.

Fifteen adult apterous *M*. *persicae* were placed on the lower surfaces of local and systemic leaves of pre-infested and uninfested plants and confined using a clip-on leaf cage (2 cm deep, 6 cm in diameter). They were allowed to reproduce for 24 h and were then removed using a small soft brush, and 20 newborn nymphs were caged on the same leaf. Survival of *M*. *persicae* was monitored daily until they matured and started reproducing offspring, generally on day 8 (the first generation). Survival was calculated from the average number of aphids per plant. After 8 d, all but two adults were removed; they were confined to a leaf clip-on cage on the lower surface of each test leaf. The fecundity was determined by counting the newborn nymphs every two days until all the adults had died; after counting, nymphs were carefully removed with a fine brush to prevent mechanical damage to the leaf surfaces. The fecundity was calculated from the average number of newborn nymphs per female aphid. Each treatment had 10 replications.

### Sampling for SA quantification

Immature whitefly-infested plants were sampled at 10, 15, and 20 d after the removal of adult whiteflies, at which points the whitefly nymphs had reached the 1^st^, 2^nd^, and 3^rd^ instar, respectively. The fourth leaf, which had 9–10 nymphs/cm^2^, and the seventh leaf, which had none, were here sampled as local and systemic leaves, respectively [36]. Leaves at the same positions on uninfested plants were sampled as controls.

Before sampling, the insects on the local leaves were gently removed with a fine brush pen. Leaves were subsequently abscised from the plants, and the main veins were removed. The leaf tissue was wrapped in aluminum foil, flash-frozen in liquid nitrogen, and stored at -20°C until needed for analysis. Three replicate plants were used for each of the three developmental stages.

### Quantification of free SA in WT and NahG plants

Free SA was extracted and quantified against an internal standard using a protocol modified from Malamy et al. [38]. Frozen leaf samples were ground into a fine power in liquid nitrogen and 0.5 g was extracted with 1 mL of 90% methanol and spiked with 2 μg/mL m-hydroxybenzoic acid (purity ≥ 99%; Pacific Market International, Seattle, WA, U.S.). After centrifugation at 4,000 rpm for 20 min at 4°C, the supernatant was collected and the pellet was extracted again with 1 mL of pure methanol. The merged supernatant was dried at 4°C in a speed vacuum concentrator, resuspended in 1 mL of 5% trichloroacetic acid and then re-extracted twice with 1 mL of ethyl acetate. The organic fractions containing free SA were collected and dried in a speed vacuum. Prior to being loaded onto an HPLC column, each sample was dissolved in 0.5 mL of methanol and filtered through a 0.22-μm filter.

HPLC separation was performed on an Agilent 1200 system (Santa Clara, CA, U.S.) equipped with a variable wavelength detector. The analytes were eluted from a C18 column (5 μm, 250 × 4.6 mm) at room temperature. The mobile phase was a binary solvent system consisting of 1% acetic acid in H_2_O (solvent A) and pure acetonitrile (solvent B) (A:B, 70:30, V:V) at a constant flow rate of 1 mL/min. SA was quantified by UV detection at 296 nm. Pure SA (purity ≥ 99%; Sigma, U.S.) was used to make the standard curve. The endogenous concentration of SA was calculated from the peak areas of the substance and its standard curve.

### Determination of defense enzymes

Experimental tobacco plants were obtained as described above. After 20 d, the local damaged leaves, systemic leaves from infested and uninfested tobacco plants (the last of which served as controls) were sampled for analysis of defense enzyme activity. Each treatment had 4 replications. All spectrophotometric analyses were conducted in a Shimadzu UV-2450 spectrophotometer (Shimadzu, Arlington, MA, U.S) at room temperature.

To assay foliar enzymes, approximately 0.2 g fresh leaf with the midribs removed was homogenized in 1 mL 0.1 M ice-cold Tris-HCl buffer, pH 7.0, containing 7% (w/v) polyvinylpolypyrollidone (PVPP; Sigma, MO, U.S.). Then, 0.4 mL 10% (v/v) Triton X-100 (Sigma) solution was added to the homogenate. The solution was centrifuged at 12,000 g for 10 min at 4°C, and the supernatant was stored at -20°C until used in spectrophotometric assays of enzymes. For the PPO assay, an aliquot of 30 μl of leaf enzyme extract was added to 1 mL of 2.92 mM caffeic acid (Sigma) in phosphoric acid buffer (0.1 M, pH 8.0) and the change in the absorbance of the mixture was measured at 398 nm for 5 min. The assay procedure for POD activities was identical, but the substrate for POD activities was 2.92 mM guaiacol (Sigma) with 0.02 mM H_2_O_2_ added as a cofactor. The change in the absorbance of the mixture was measured at 470nm. PPO and POD both indicate the rate of formation of melanin-like material from phenolic substrates [39]. The activities of PPO and POD are reported variations in as optical density (OD) variation per min per gram fresh weight sample.

The β-1,3-glucanase assay was performed using the method described by Abeles and Forrence et al. [40]. This method involved using laminarin (Sigma) as a substrate and the dinitrosalicyclic (Sigma) reagent was used to measure the reducing sugars produced. We routinely added 100 μl of plant extract diluted to 0.5 mL of 1% (w/v) laminarin in water and the mixture was incubated at 37°C for 30 minutes. The laminarin solution was heated briefly in a boiling water bath before use. The reaction was stopped by adding 1 mL of the dinitrosalicylic reagent and the tubes were heated for 5 min at 100°C. The tubes were then cooled to 25°C, and the optical density was read at 540 nm. The β-1,3-gluanse activity was defined as μmol glucose equivalents released per min per gram fresh weight.

The chitinase assay was performed using the method presented by Sawborowski et al. [41]. Utilizing glycol chitin (Sigma) as a substrate, 100 μl of plant extract diluted in 0.05 M (pH 8.0) phosphate buffer was added to 500 μl of 1% glycol chitin (Sigma) and incubated at 37°C for 1h. The reaction was stopped by adding 1.5 mL of dinitrosalicyclic (Sigma) and then boiled for 5 min. The tubes were then cooled to 25°C. The absorbance was measured at 520 nm. The chitinase activity was defined as μmol N-acetylglucosamine equivalents released per hour per gram fresh weight.

### Statistical analysis

Data were analyzed using the statistical software package SPSS version 18.0 (SPSS, Chicago, USA). *M*. *persicae* survival has been tested with a Cox proportional hazards model. Significant differences in the amounts of SA and defense enzymes activities of tobacco leaves were tested by a mixed effect model and factorial ANOVA model using Tukey’s test at a significance level of 5% (*P* < 0.05).

## Results

### Survival of *M*. *persicae* on WT and NahG plants infested with whitefly nymphs

**Fig 1:**
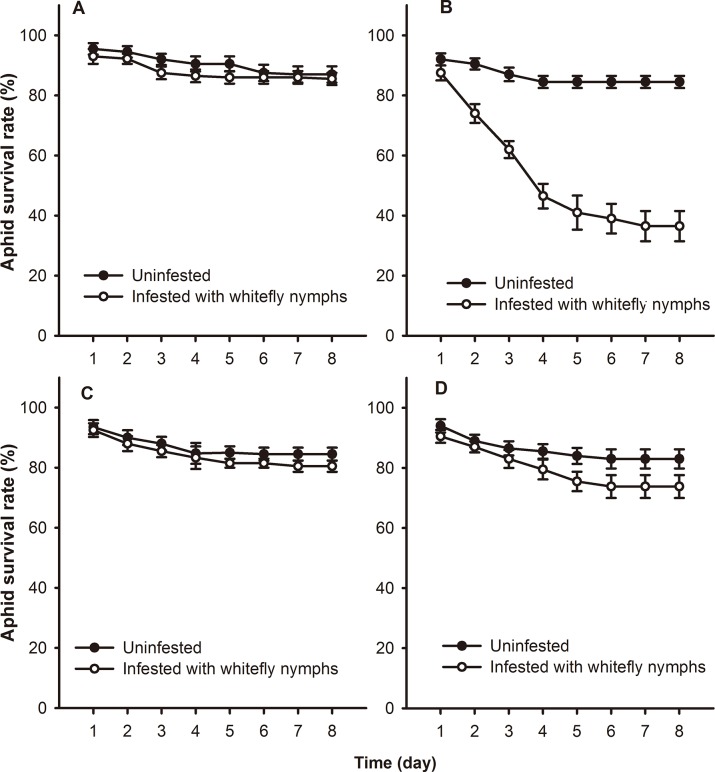
Survival of *M*. *persicae* on *B*. *tabaci* nymph-infested tobacco plants. *M*. *persicae* survival on (A) local and (B) systemic leaves of WT tobacco plants. *M*. *persicae* survival on (C) local and (D) systemic leaves of NahG tobacco plants. Values represent the mean survival of aphids ± standard error.

Based on Cox proportional hazards model, survival of *M*. *persicae* on local leaves of infested WT plants did not differ significantly from that of the uninfested control (*P* = 0.859) (Fig 1A), but it was significantly lower than the control values on systemic leaves (*P* < 0.001). Survival was 46.9% lower (*F* = 1.344; *df* = 1,18; *P* < 0.001) on the systemic leaves of plants infested with *B*. *tabaci* nymphs than on controls (Fig 1B). No significant difference was found in survival between *B*. *tabaci* pre-infested NahG and uninfested control plants (Fig 1C and 1D).

### Fecundity of adult *M*. *persicae* on WT and NahG plants infested with whitefly nymphs

**Fig 2:**
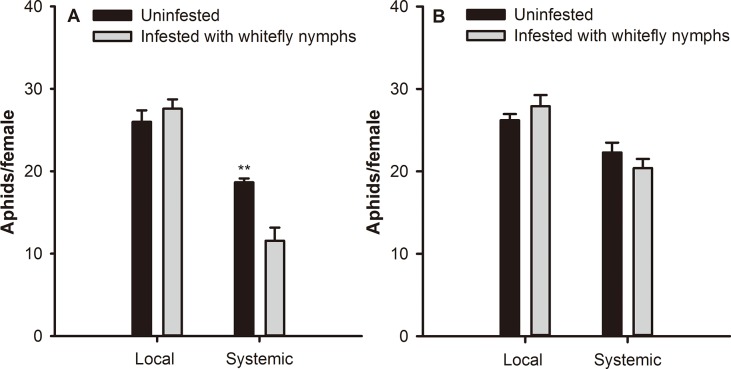
Fecundity of *M*. *persicae* on *B*. *tabaci* nymph-infested tobacco plants. *M*. *persicae* fecundity on local and systemic leaves of (A) WT and (B) NahG tobacco plants. Values represent the mean number of newborn nymphs per female ± standard error. ^**^ indicates significant differences (*P* ≤ 0.01) relative to the controls.

Based on mixed effects model, whitefly infestation (*F* = 4.882; *df* = 1,37; *P* < 0.05) and leaf position (*F* = 74.6; *df* = 1,37; *P* < 0.001) had significant effects on fecundity of *M*. *persicae* in WT plants. In local leaves, fecundity of *M*. *persicae* was not significantly affected by *B*. *tabaci* nymph infestation (*F* = 37.578; *df* = 1,39; *P* = 0.832) (Fig 2A). In systemic leaves, however, fecundity was 38.0% lower than the uninfested control (*F* = 37.5; *df* = 1,39; *P* < 0.001). In both local and systemic leaves, no difference was found in the fecundity of the *B*. *tabaci* nymph pre-infested NahG and uninfested control plants (Fig 2B).

### Quantification of SA levels in WT and *NahG* plants infested with whitefly nymphs

**Fig 3:**
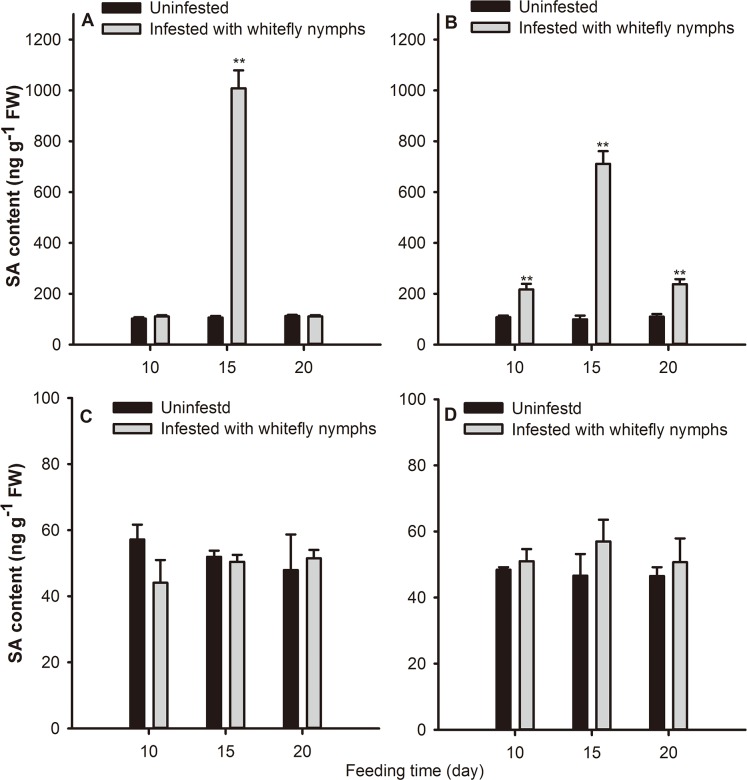
Quantification of SA hormone levels in *B*. *tabaci* nymph-infested tobacco plants. SA levels in (A) local and (B) systemic leaves of WT plants and in (C) local and (D) systemic leaves of NahG plants after 10, 15, and 20 days of *B*. *tabaci* nymphs infestation. Values represent the mean ng SA per g fresh weight (FW) ± standard error. ^**^indicates significant differences (*P* ≤ 0.01) relative to the controls.

Whitefly infestation (*F* = 26.355; *df* = 1,41; *P* < 0.001) and feeding time (*F* = 15.853; *df* = 2,41; *P* < 0.001) both had significant effects on the SA content in WT plants in the mixed effects model. After *B*. *tabaci* nymph infestation, significantly higher levels of SA were detected in local and systemic leaves of WT plants than in uninfested control plants. The SA content of nymph-infested leaves was 8.45-fold higher (*F* = 157.398; *df* = 1,23; *P* < 0.001) than the uninfested control after 15 d of infestation (Fig 3A), while the SA content was 6.14-fold higher in systemic leaves (*F* = 81.362; *df* = 1,21; *P* < 0.001) (Fig 3B). Although a decreasing trend was observed in the SA content 20 d after infestation, this value was 1.16-fold higher in systemic leaves than in the uninfested control (*F* = 81.362; *df* = 1,21; *P* < 0.05) (Fig 3B). Whitefly infestation (*F* = 0.035; *df* = 1,28; *P* = 0.854) and feeding time (*F* = 0.079; *df* = 2,28; *P* = 0.924) had no significant effect on the SA content in NahG plants. The SA content did not differ between infested NahG and uninfested control plants (Fig 3C and 3D).

### Whitefly nymphs infestation on defense enzyme activity levels in WT and NahG tobacco plants

Plant type (*F* = 24.685; *df* = 1,28; *P* < 0.001) had significant effects on PPO activities in the mixed effects model, but leaf position did not (*F* = 0.625; *df* = 1,28; *P* = 0.436). In WT plants, *B*. *tabaci* nymphs infestation rendered the activity of PPO 54.2% higher than in controls locally (*F* = 30.511; *df* = 1,15; *P* = 0.001) and 48.1% higher than in controls systemically (*F* = 30.511; *df* = 1,15; *P* < 0.001). The PPO activity in infested and systemic leaves of NahG plants did not differ from control values.

Leaf position (*F* = 13.210; *df* = 1,28; *P* = 0.001) had significant effects on POD activities in tobacco leaves, but plant type did not (*F* = 3.066; *df* = 1,28; *P* = 0.094). In WT plants infested with *B*. *tabaci* nymphs, POD activity was 47.9% higher than in controls in local leaves and 55.8% higher than in controls in systemic leaves (*F* = 36.357; *df* = 1,15; *P* < 0.001) (Table 1). Similarly, in NahG plants POD was 43.5% higher than in controls in local leaves (*F* = 25.753; *df* = 1,15; *P* < 0.001) and 36.1% higher in systemic leaves (*F* = 25.753; *df* = 1,15; *P* = 0.001) (Table 1).

**Table 1:** Infestation by *B*. *tabaci* nymphs on defense enzyme activity levels of WT and NahG tobacco plants.

	Polyphenol oxidase		Peroxidase		β-1,3-glucanase		Chitinase	
	(OD398 min^-1^ g^-1^ FW)		(OD470 min^-1^ g^-1^ FW)		(μmol min^-1^g ^-1^ FW)		(μmol h^-1^ g^-1^ FW)	
Treatment	WT	NahG	WT	NahG	WT	NahG	WT	NahG
**Local Infested**	0.63±0.04A,a	0.45±0.02A,b	21.4±1.2B,a	21.7±0.7A,a	21.5±0.5B,a	15.4±0.9A,b	146±2.9B,a	102±3.1B,b
**Local Control**	0.41±0.02B,a	0.41±0.01A,a	14.4±0.8C,a	15.1±0.6B,a	16.7±0.9C,a	15.8±1.5A,a	117.5±2.5C,a	119.8±2.4A,a
**Systemic Infested**	0.66±0.01A,a	0.46±0.01A,b	26.8±0.3A,a	22.1±0.8A,b	27.4±1.8A,a	18.4±1.6A,b	157.5±4.4A,a	106.3±3.0A,b
**Systemic Control**	0.45±0.01B,a	0.41±0.01A,a	17.2±1.0C,a	16.2±0.7B,a	15.9±1.3C,a	15.5±0.7A,a	115.5±4.0C,a	104±2.7aB,b

Each value represents the average (± SE) of four replicates. The different capital letters in each of the column indicate significant differences among local and systemic leaves of treated plants in activity levels of each defense enzyme (*P* < 0.05). The different lowercase letters at each of the rows indicate significant differences between WT and NahG plants in activity levels of each defense enzyme (*P* < 0.05).

Plant type (*F* = 12.025; *df* = 1,28; *P* = 0.002) had significant effects on β-1,3-glucanase activities in tobacco leaves, but leaf position did not (*F* = 2.675; *df* = 1,28; *P* = 0.113). In WT plants, β-1,3-glucanase activity differed greatly between those infested with *B*. *tabaci* nymphs and the control and was significantly higher than controls, by 28.3%, locally (*F* = 15.016; *df* = 1,15; *P* < 0.05) and 72.1% higher than in controls systemically (*F* = 15.016; *df* = 1,15; *P* < 0.001) (Table 1). In addition, the activity of β-1, 3-glucanase was much higher in systemic leaves than in local leaves. In NahG tobacco plants, the β-1,3-glucanse activity of local and systemic leaves was not statistically different from the control values.

Plant type (*F* = 43.646; *df* = 1,28; *P* = <0.001) had significant effects on chitinase activities in tobacco leaves, but leaf position did not (*F* = 0.213; *df* = 1,28; *P* = 0.648). *B*. *tabaci* nymph infestation increased the chitinase activity by 24.6% locally (*F* = 35.364; *df* = 1,15; *P* < 0.001) and 36.3% systemically (*F* = 35.364; *df* = 1,15; *P* < 0.001) in WT plants (Table 1). In addition, the chitinase activity was much higher in systemic leaves than in local leaves; however, the chitinase activity in different leaves of NahG plants was not statistically different from that of control leaves.

## Discussion

Plants deploy defense-signaling pathways, which are regulated by different hormones, in response to biotic stress [42, 43]. Salicylic acid is an endogenous signal substance ubiquitous in plants. It plays a crucial role in plant defense against pathogens and herbivores [12, 16, 44]. Previous researches with *Arabidopsis* have shown that after *B*. *tabaci* feeding, levels of SA-regulated host genes or proteins increase both locally and systemically, while expression levels of JA- and ET-regulated genes or proteins decline [9, 31, 32]. Here, we investigate the role of the salicylate pathway in defense induced by *B*. *tabaci* against *M*. *persicae* in tobacco plants. Our performance results showed that *B*. *tabaci* infestation systemically rendered *M*. *persicae* survival and fecundity lower in wild-type-plants than in control plants; but infestation had no visible results in salicylate deficient NahG plants, which indicates a salicylate-dependent mechanism underlies this induced resistance. These findings are in line with previous studies showing that aphids survive longer on tomato plants ectopically expressing NahG than on wild-type plants [17, 45, 46], and that the application of BTH reduces the growth of aphids [47].

Plants accurately regulate the signaling pathways by adjusting signal substance levels [48]. *B*. *tabaci* nymphs induced the accumulation of SA in WT plants, 5 times more than in the control in local and systemic leaves indicating that *B*. *tabaci* nymphs could induce the SA defense signal pathway. Further, we found the SA content varies depending on the feeding stage of *B*. *tabaci*, and was positively correlated with systemic defense against *M*. *persicae*. SA content peaked 15days after infestation, but it returned to control levels on day 20. This trend is consistent with our previous data, i.e., that the resistance to aphids induced by *B*. *tabaci* was most obvious at the third instar [49]. Similarly, Estrada-Hernandez et al. [50] indicated that SA biosynthesis-related genes in tomato were induced during the early stages of *B*. *tabaci* (biotype A) infestation but were later repressed. Most of the defense gene expression modifications were detected in the second to third nymphal transition instar. In this way, resistance to aphids was found to be associated with high levels of SA.

It is here concluded that the introduction of NahG may abolish resistance to aphids. Interestingly, in WT plants, *B*. *tabaci* infestation induced an obvious increase in SA content over control in local leaves but there was no change in survival or fecundity of subsequent *M*. *persicae*. This indicates that SA does not function in direct defense against *M*. *persicae* but it does specifically induce systemic defense responses. Inbar et al. [22] found that the defense effect on *Liriomyza trifolii* induced by *B*. *tabaci* infestation which varied between local and systemic leaves, was closely related to the activities of defense enzymes.

SA can transmit signals to plants, leading to increases in defense proteins and secondary metabolites that act as feeding deterrents, anti-nutritional factors, or toxins [13, 15]. Defense proteins such as PPO, POD, β-1,3-glucanase, and chitinase are considered to play key roles in SA-mediated defense responses [51–53]. In this study, the magnitude of the response to each enzyme was differently affected by *B*. *tabaci* nymph infestation. In WT plants, the activities of PPO, POD, β-1,3-glucanase, and chitinase were significantly induced, both locally and systemically by *B*. *tabaci* nymph infestation. However, no significant difference in the activities of β-1,3-glucanase and chitinase was observed between NahG and control plants. This shows that *B*. *tabaci*-induced β-1,3-glucanase and chitinase changes depended on SA, whereas POD was not involved in this SA-dependent resistance, although it can catalyze the sclerotization of plant cell walls, which creates a physical barrier that limits pathogen invasion and possibly aphid feeding [54]. More importantly, SA mediated defense enzymes were differently affected in different leaf positions of WT plants. β-1,3-glucanase and chitinase activities were much higher in systemic than in local leaves. However, PPO showed the opposite pattern. Phloem feeders such as whiteflies and aphids may deliver effectors inside their hosts to manipulate defense response enabling successful infestation of plants. Because *B*. *tabaci* nymph infestation can induce a systemic but not local resistance to *M*. *persicae*. It is here believed that β-1,3-glucanase and chitinase activities may be positively correlated with the resistance to aphids. β-1,3-glucanase and chitinase were involved in the defense against Russian wheat aphids in wheat [55] and *Liriomyza trifolii* in tomato [22] plants. Chitinases may interfere with insect development, feeding, and growth and facilitate microbial infection by damaging the chitin-based peritrophic membrane that acts as a primary barrier to pathogen infection [56, 57]. However, the role of β-1,3-glucanase in insect resistance remains poorly understood. One possible mechanism for β-1,3-glucanase action is to release oligosaccharides from the plant cell wall; these are molecules that are known to trigger other defense reactions in plants [58].

In general, induced resistance to herbivore damage ranges over spatial scales from single leaves to whole plants [59], while leaves located at different positions on a plant may differ in their responses to herbivores [60, 61]. Previous research has indicated that the defense response and adverse impact of different leaf positions of plants pre-infested with phloem-feeders or other herbivores were diverse. For example, a transcript encoding phloem protein 2 lectin (PP2-A7), proposed to function in aphid defense, had a 5-fold higher expression level in infested leaves and a 22-fold higher expression level in systemic leaves than equivalent control leaves in *Arabidopsis* [62, 63]. Dugravot et al. [64] found *M*. *persicae* food acceptance was slightly enhanced on potato leaves previously infested by *M*. *persicae* or by heterospecific *Macrosiphum euphorbiae*, whereas food acceptance was inhibited on the uninfested leaves of infested plants, which was similar to the present results. Insect saliva plays an important role in plant induced defense [30, 65]. It is here speculated that there may be some specific components in insect saliva that could weaken the local defense. The induction of plant defenses and their subsequent suppression by insects is thought to be an important factor in the evolutionary arms race between plants and herbivores. For example, protein factor Mp10 in *M*. *persicae* saliva reduces induced tobacco PTI defenses, and overexpression of the saliva C002 gene rendered the transgenic tobacco plants a more suitable host for aphids [66].

Induced plant responses can mediate interspecific competition among the arthropods that share the same host plants [22]. In this study, feeding of *B*. *tabaci* decreased the performance of *M*. *persicae* in systemic leaves of tobacco plants via increased salicylate signaling. In addition, previous works showed that *B*. *tabaci* can manipulate plant-induced resistance and is able to suppress effective JA/ET defenses through the induction of the SA signaling-based responses [31, 32, 67]. All these findings indicate that specific induced defense allow *B*. *tabaci* to not only defeat other competitors but evade effective defense, offering them an advantage in interspecific competition.
